# Solution-Processed Inorganic Perovskite Flexible Photodetectors with High Performance

**DOI:** 10.1186/s11671-019-3120-x

**Published:** 2019-08-17

**Authors:** Ziji Liu, Hao Li, Chaojie Qin, Ting Zhang, Yiding Gu, Hao Chen, Hualin Zheng, Shibin Li

**Affiliations:** State Key Laboratory of Electronic Thin Films and Integrated Devices, and School of Optoelectronic Science and Engineering, University of Electronic Science and Technology of China (UESTC), Chengdu, 610054 Sichuan China

**Keywords:** CsPbI_3-x_Br_x_-based flexible PDs, Low bias voltage, Environmental stability, Mechanical flexibility

## Abstract

All inorganic CsPbI_3-x_Br_x_ perovskites have been widely used in photodetectors due to their excellent optoelectronic properties and simple preparation processes. Here, high-performance flexible photodetectors based on inorganic CsPbI_3-x_Br_x_ perovskites are demonstrated, which are achieved by a modified solution-processed method. When biased at a low voltage of 10 mV, the device yielded fast response speeds (90 μs /110 μs for CsPbI_2_Br PDs and 100 μs/140 μs for CsPbIBr_2_ PDs), a high on/off ratio of 10^4^, and a high detectivity about 10^12^ Jones. Meanwhile, the devices showed outstanding environmental stability and mechanical flexibility. The periodic I-t curves had negligible fluctuation (< 5%) after storing in air atmosphere for 30 days or bending for 100 times. The results indicate that CsPbI_3-x_Br_x_ perovskites have great potential in photodetection areas and pave the way to achieve high-performance flexible PDs.

## Introduction

In the past few decades, a variety of inorganic semiconductor materials have drawn most attention of photodetectors research, such as InGaAs, GaN, ZnO, and Si [1–6]. Benefited by their excellent optical and electrical properties, the devices based on these materials exhibit high detectivity and fast response for visible light. However, such materials are usually obtained by extremely complex approaches or using expensive equipment [7–9], which is a bottleneck on the way to their commercial application. Hence, seeking for more promising substitute materials to reduce the cost, and simplify the preparation process is very important.

Recently, hybrid halide perovskites (HHPs) materials have been one of the research hot spots in the field of photovoltaic devices [10–15]. In the last decade, the power conversion efficiencies of perovskite solar cells have been increased from 3.8% to exceed 23% [16–27], owing to its remarkable optoelectronic properties, such as the merits of the optimum bandgap, high absorption coefficient, and superior ambipolar carrier transport ability [28–31]. In addition, low-cost and simple solution process preparation also makes perovskites have great potential in the research of photodetectors. However, in spite of the photovoltaic devices based on HHPs have made rapid and impressive progress, they still suffer from poor stability [32, 33]. Compared with HHPs, inorganic cesium lead halide perovskites (IHPs) exhibit better stability in the air, indicating that IHPs is an ideal candidate for photodetectors [34, 35]. Although, CsPbI_3_ is unstable at high temperature (above 300 °C), which can be improved by substituting the Br^−^ moiety for I^−^ [36–41]. Therefore, CsPbI_3-x_Br_x_ is very suitable for designing high-performance photodetectors.

In this work, we prepared CsPbI_3-x_Br_x_ (*x* = 1, 2) films for flexible perovskite photodetectors (PDs). The flexible perovskite PDs based on CsPbI_3-x_Br_x_ exhibited fast response speed (90 μs/110 μs for CsPbI_2_Br PDs and 100 μs/140 μs for CsPbIBr_2_ PDs), a high on/off ratio (10^4^), and a high detectivity (10^12^ Jones) under 520 nm lamp with 10 mV bias. Meanwhile, it exhibited outstanding mechanical flexibility and environmental stability. After keeping the device for 30 days in ambient air at 35–45% relative humidity, the periodic I-t curves of the device are only slightly declined (~ 3%). In addition, after bending the flexible PDs for 100 times under the bending radius of 9.12 mm, the periodic I-t curves of the device showed negligible change (< 3% decrease). The results indicate the potential of CsPbI_3-x_Br_x_ perovskites for flexible PDs.

## Method

### Materials

Lead iodide (PbI_2_, 99.99%), lead bromide (PbBr_2_, 99.99%), barium iodide (CsI, 99.99%), and barium bromide (CsBr, 99.99%) were purchased from Xi’an Polymer Light Technology Corporation. Diethyl ether (DEE), acetone, absolute ethanol, *N*,*N*-dimethylformamide (DMF), and dimethylsulfoxide (DMSO) were provided by Sigma-Aldrich.

The perovskite solution was prepared as follows. The CsPbI_2_Br perovskite precursor solutions were prepared by mixing the 1 M (mol L^−1^) CsBr, 1 M PbBr_2_, 2 M CsI, and 2 M PbI_2_ in mixed anhydrous solvents of DMSO and DMF (9:1 in volume). The CsPbIBr_2_ perovskite precursor solutions were prepared by mixing the 2 M CsBr, 2 M PbBr_2_, 1 M CsI, and 1 M PbI_2_ in mixed anhydrous solvents of DMSO and DMF (9:1 in volume). Then, the mixed perovskite solutions were stirred at 75 °C more than 2 h. All solutions must be were prepared in a nitrogen glovebox.

### Preparation

Flexible substrates [polyimide (PI)] were cleaned consecutively with acetone, absolute ethanol, and deionized water for 15 min, respectively. And then the substrates were dried in an oven. Subsequently, interdigitated golden electrode (80 nm) was evaporated onto the flexible substrates by thermal evaporation. Before use, the patterned substrates were treated with UV-ozone for 20 min. Then the flexible substrates were transferred into glovebox for perovskite films deposition. The CsPbI_3-x_Br_x_ films were prepared using anti-solvent (DEE) one-step spin-coating method. Eighty-microliter precursor solution was spin-coating at a speed of 2000 rpm for 60 s. Then, 0.5 mL diethyl ether was poured on the samples at 10 s before the end of the spinning-coating processes. Then, the samples were annealed at 65 °C for 5 min and 135 °C for 15 min.

### Measurements and Characterizations

Scanning electron microscope (SEM) images were obtained by using field emission SEM (FEI- INSPECT F50, Holland). X-ray diffraction (XRD) was carried out using a Bede D1 system with Cu Kα radiation. The ultraviolet-visible (UV-vis) absorption spectrum was measured by an UV-vis spectrophotometer (Schimadzu UV-3101 PC). The current–voltage (*I-V*) curves were carried out by Keithley 2636 semiconductor parametric analyzer under the illumination of a LD light source. Photocurrent was measured with an oscilloscope (Agilent DOS5012A) and an optical chopper modulating the light illuminated on the device. All measurements were carried out under ambient conditions at room temperature.

## Results and Discussion

**Fig. 1:**
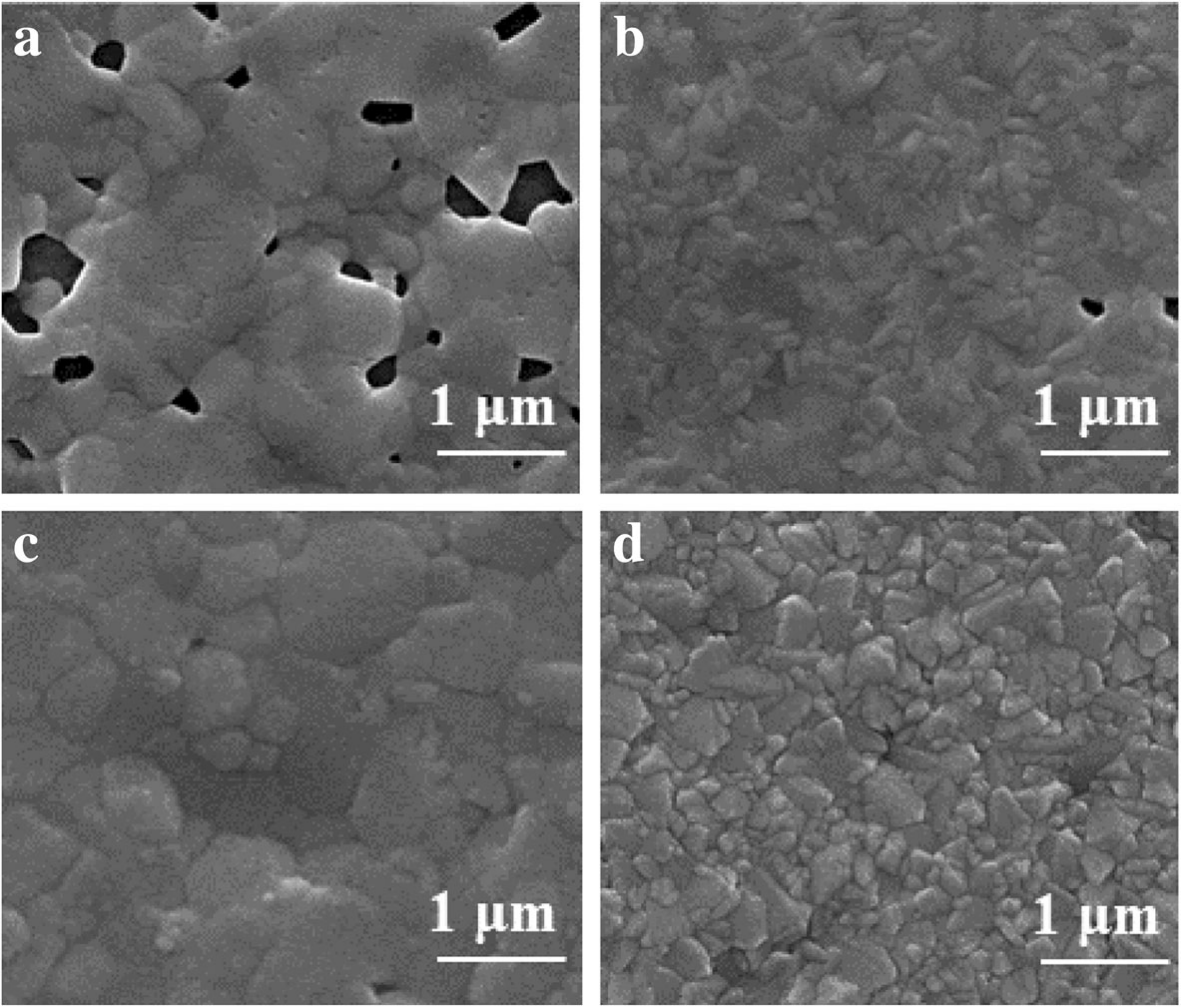
SEM image of the CsPbI_3-x_Br_x_ films. **a** CsPbI_2_Br, **b** CsPbIBr_2_ without DEE treatment and **c** CsPbI_2_Br, **d** CsPbIBr_2_ with DEE treatment

Anti-solvents, which are often used in the preparation of perovskite films to obtain a high-quality surface morphology. Herein, we used one-step spin-coating method with anti-solvent (DEE) to improve the morphology of CsPbI_3-x_Br_x_ films. Figure 1 exhibits the plan-view SEM images of CsPbI_3-x_Br_x_ films with or without DEE treatment. As shown in Fig. 1a, b, the CsPbI_2_Br and CsPbIBr_2_ film without DEE-treatment possess enormous pinholes and small crystal grain size. In contrast, after DEE-treatment in the fabrication of perovskite, continuous film and larger grains are observed in the SEM images (Fig. 1c, d). The results of SEM images indicated that the morphology of the CsPbI_3-x_Br_x_ films was significantly improved by the treatment of DEE. Therefore, in the subsequent work, the anti-solvent treatment process was adopted to prepare CsPbI_3-x_Br_x_ films.

**Fig. 2:**
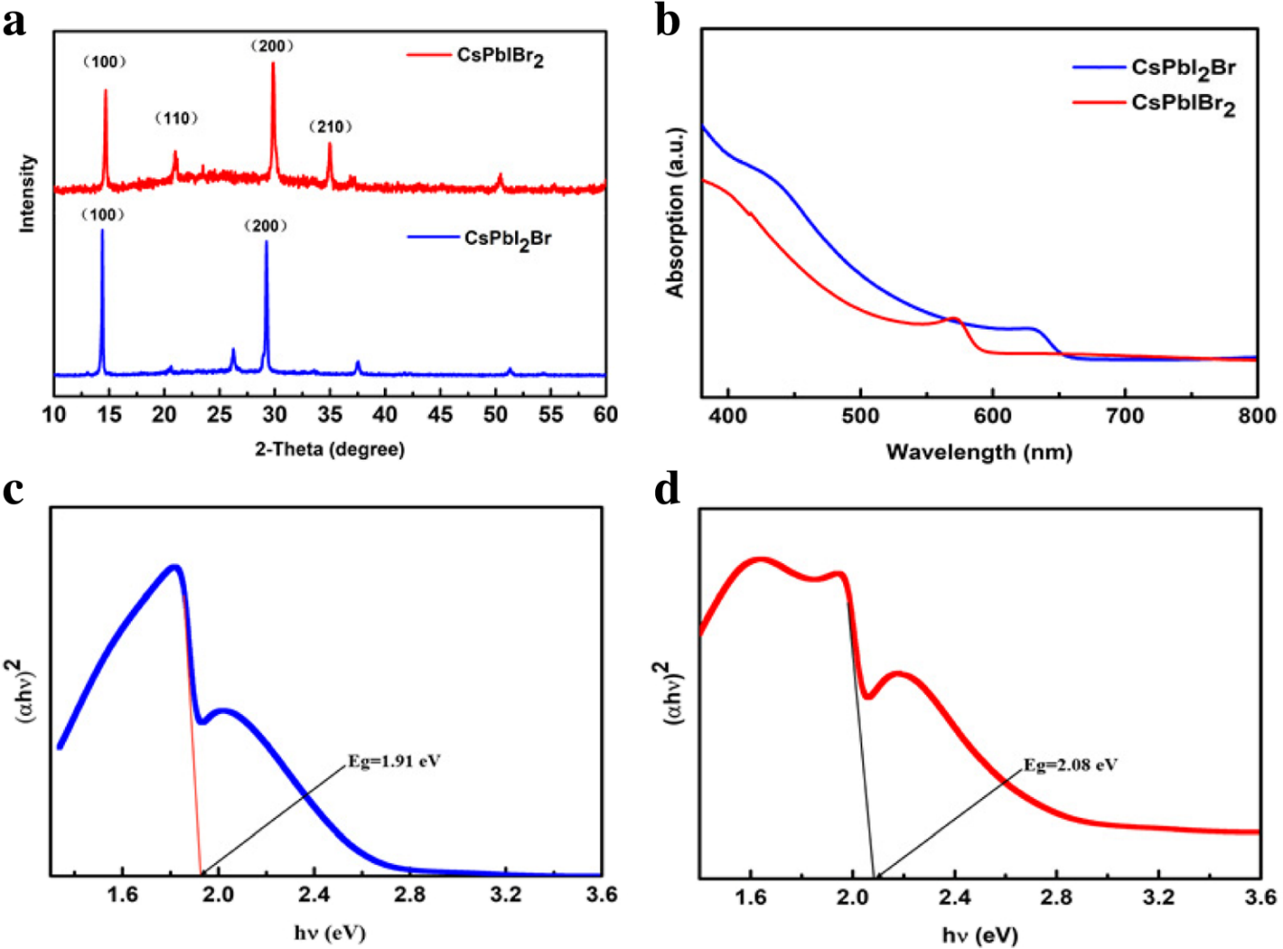
**a** XRD patterns. **b** Absorption of the CsPbI_3-x_Br_x_ films. The band gap of **c** CsPbI_2_Br and **d** CsPbIBr_2_

In order to examine the crystal structure of the prepared CsPbI_3-x_Br_x_ films, XRD patterns were carried out. As shown in Fig. 2a, the red and the blue lines indicate the XRD patterns of CsPbIBr_2_ films and CsPbI_2_Br, respectively. It was clear to observe, in the red lines, the main peaks located at 14.75°, 20.94°, 29.96°, and 34.93°, which are assigned to the (100), (110), (200), and (210) facts of CsPbIBr_2_, respectively [41–43]. In the blue lines, we note two main peaks centered at 14.44° and 20.3° corresponding to the (100) and (200) planes of the pure CsPbI_2_Br α phase [44, 45]. In addition, to certify that the CsPbIBr_2_ and CsPbI_2_Br films were successfully prepared, UV-vis absorption curves and Tauc plots were measured (Fig. 2b–d). As shown in Fig. 2b, a small blue shift is visible in the absorption spectrum of the CsPbIBr_2_ film compared to the CsPbI_2_Br film, which attributed to the bandgap difference between CsPbI_2_Br and CsPbIBr_2_. To explore the optical bandgap of CsPbI_3-x_Br_x_ film, we calculated the energy band (Eg) according to the reflection and transmission through the Tauc plots, as shown in Fig. 2c, d. It was observed from the Tauc plots that Eg of CsPbI_2_Br and CsPbIBr_2_ were 1.91 eV and 2.05 eV, respectively, which is consistent with the previous report [46–50]. The results indicated the CsPbI_3-x_Br_x_ films were successfully fabricated.

**Fig. 3:**
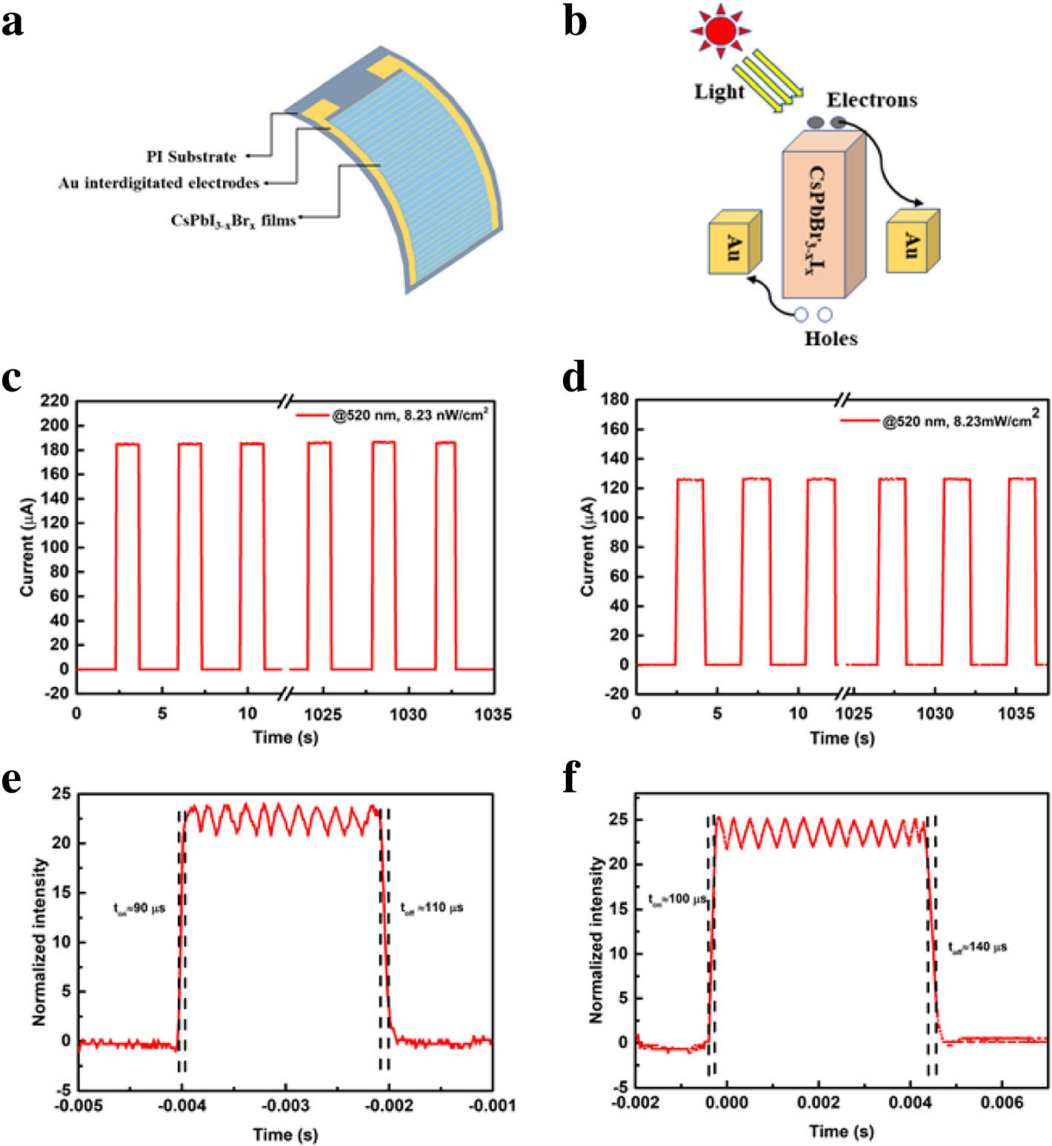
**a** Device structure of the CsPbI_3-x_Br_x_ flexible PDs**. b** Schematic diagram of charge carrier transport in the device under illumination. I–t curves of the **c** CsPbI_2_Br PDs and **d** CsPbIBr2 PDs upon 520 nm light at a bias of 10 mV. The rise time (ton) and fall time (toff) of **e** CsPbI_2_Br PDs and **f** CsPbIBr_2_ PDs, respectively

After confirming the properties of the treated CsPbI_3-x_Br_x_ films, we prepared the flexible PDs based on CsPbI_3-x_Br_x_ films. In Fig. 3a, the flexible PDs with the device structure of PI/Au interdigitated electrodes/CsPbI_3-x_Br_x_ are shown. When it is irradiated on the perovskite layer under a bias voltage, the charge carrier transport is formed in PDs, as shown in Fig. 3b. Finally, electrons (holes) are collected by electrodes and circulated through an external circuit to generate photocurrent. To characterize the performance of CsPbI_3-x_Br_x_ PDs, we measured the photocurrent under 520 nm LD source at low bias voltages as shown in Fig. 3c, d. The result showed that the maximum photocurrent of CsPbI_2_Br PDs and CsPbIBr_2_ PDs were beyond 180 μA and 120 μA under the illumination intensity of 8.23 mW/cm^2^, respectively. Importantly, the photocurrent of the device was measured at a bias voltage of 10 mV. To evaluate the response speed of the device, we investigated the transient photocurrent of the device. As shown in Fig. 3c, d, the rise and decay time of CsPbI_2_Br PDs are extracted to be around 90 μs and 110 μs, respectively. The rise and decay time of CsPbIBr_2_ PDs were found to be about 100 μs and 140 μs, respectively. Compared to the same structural devices previously reported [12], the results manifested that the device based on DEE-treated CsPbI_3-x_Br_x_ perovskite layer showed excellent performance.

**Fig. 4:**
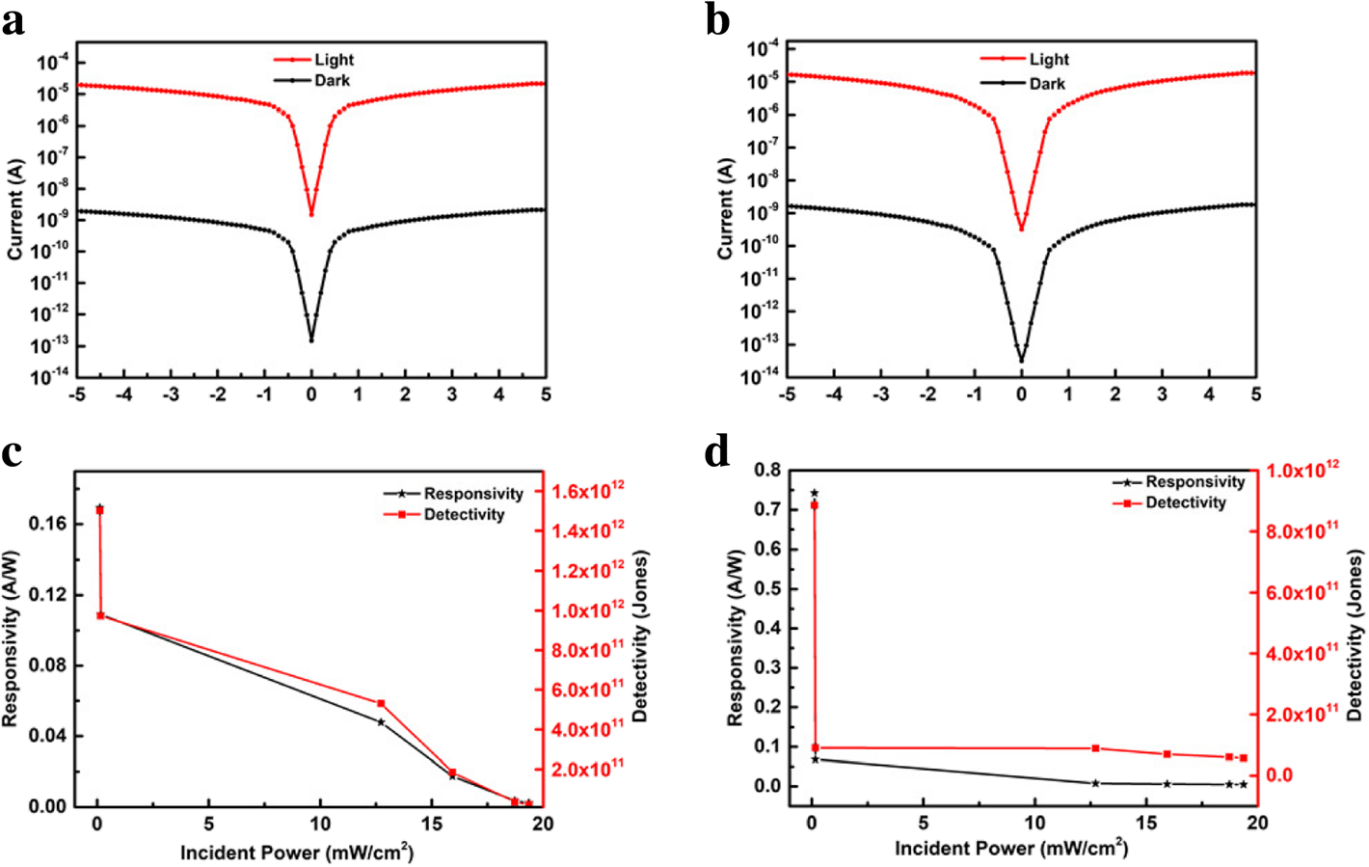
The curves (I-V) of **a** CsPbI_2_Br PDs and **b** CsPbIBr_2_ PDs for photocurrent (520 nm LD) and dark current. The responsivity and detectivity (illustration) of the **c** CsPbI_2_Br PDs and **d** CsPbIBr_2_ PDs (520 nm LD) under 10 mV voltage

To further investigate the photoelectrical characteristics of the PDs, the dark current and photo-current, responsivity, and detectivity were measured and plotted, as shown in Fig. 4. As showed in Fig. 4a, b, the dark current and photocurrent curves have approximate symmetry when the voltage was changed from − 5 to 5 V. This result indicated the formation of an ohmic contact between the metal and the perovskite layer. Owing to the contact barrier being very low for ohmic contact, carriers can be easily transferred under low bias. At low bias voltages, the CsPbI_3-x_Br_x_ flexible PDs possess a high on/off ratio of approximately 10^4^. Responsivity (*R*) and detectivity (D*) of the PDs were showed in Fig. 4c, d. The image illustrated that the *R* and D* had high values under weak light at low bias, especially below 1 mW, along with a high D* of 10^12^ Jones@10 mV bias. Zhang et al. reported a CsPbBr_3_-based flexible PDs with a D* of 10^10^ Jones under 2 V bias [12]. Ding et al. reported a rigid PDs based on CsPbBr_3_ single crystals with a D* of 10^11^ Jones [51]. Therefore, the CsPbI_3-x_Br_x_-based flexible PDs working under low bias voltages exhibit outstanding performances.

**Fig. 5:**
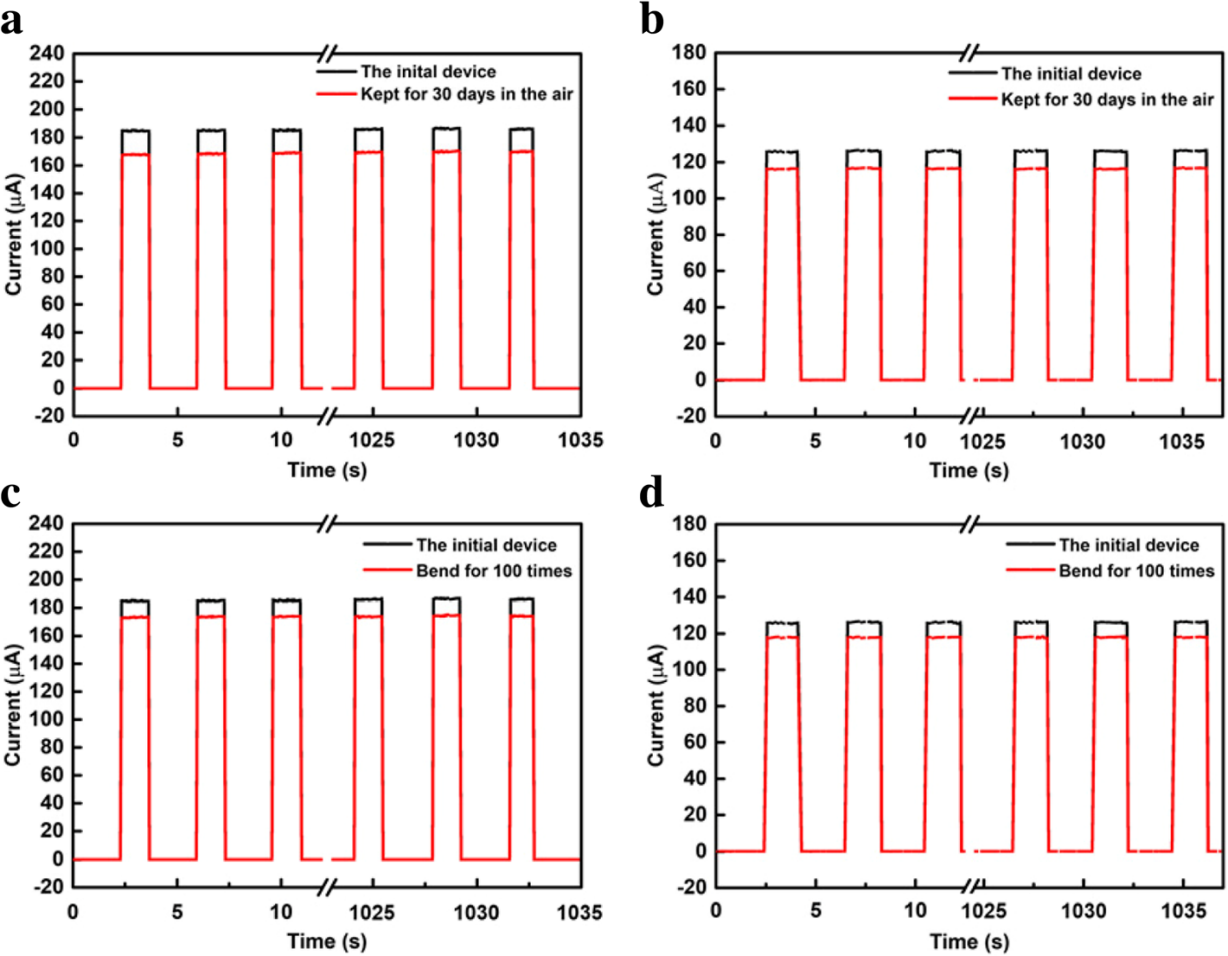
Comparison of the reproducible I-t curves of **a** CsPbI_2_Br PDs and **b** CsPbIBr_2_ PDs kept in air for 30 days. Comparison of the reproducible I-t curves of **c** CsPbI_2_Br PDs and **d** CsPbIBr_2_ PDs bending for 100 times

Environmental stability and mechanical flexibility are two important factors that influence the practical application of photodetectors based on perovskite. As described in Fig. 5, the environmental stability and mechanical flexibility of the PDs were tested by measuring the changes in periodic I-t curves of the device. It was clearly observed that the I-t curves of the device changed slightly (~ 3% decrease for CsPbI_2_Br PDs and ~ 3% decrease for CsPbIBr_2_ PDs). The changes of the devices’ currents could be ignored after being exposed for 30 days in ambient conditions at 35–45% relative humidity (Fig. 5a, b). As showed in Fig. 5c, d, compared with the initial periodic I-t curves, a negligible oscillation was observed (~ 2% decrease for CsPbI_2_Br PDs and ~ 3% decrease for CsPbIBr_2_ PDs) after bending the device for one hundred times under the bending radius of 9.12 mm. The results demonstrate that our device has a great stability as well as good mechanical flexibility.

## Conclusion

In summary, we present the flexible photodetectors based on CsPbI_3-x_Br_x_ (*x* = 1, 2) films which were treated with DEE in this work. The devices showed excellent performance, which were comparable to the same configuration devices. At a bias of 10 mV, the photodetectors showed a high on/off ratio of 10^4^ under 520 nm illumination, fast response speed (90 μs/110 μs for CsPbI_2_Br PDs and 100 μs/140 μs for CsPbIBr_2_ PDs), and an excellent detectivity (10^12^ Jones). Furthermore, the flexible PDs exhibited outstanding environmental stability and mechanical flexibility. After storing the device for 30 days in air at 35–45% relative humidity, the I-t curves of the device are only slightly declined (~ 3%). In addition, performance of the PDs showed a negligible change after bending the flexible PD a hundred times with a bend radius of 9.12 mm. This work demonstrates the enormous potential of CsPbI_3-x_Br_x_ perovskites in photoelectron detection and provides a promising approach to achieving high performance.

## Data Availability

The datasets used and/or analyzed during the current study are obtained from the corresponding author on reasonable request.

## Abbreviations

DEE: Diethyl ether
DMF: *N*,*N*-dimethylformamide
DMSO: Dimethylsulfoxide
HHPs: Hybrid halide perovskites
IHPs: Inorganic cesium lead halide perovskites
PDs: Photodetectors
SEM: Scanning electron microscope
UV-vis: Ultraviolet-visible
XRD: X-ray diffraction
